# The Preference, Effect, and Prognosis of Intra-Aortic Balloon Counterpulsation in Acute Myocardial Infarction Complicated by Cardiogenic Shock Patients: A Retrospective Cohort Study

**DOI:** 10.1155/2021/6656926

**Published:** 2021-01-20

**Authors:** Wenjun Wang, Feifei Yang, Xixiang Lin, Qin Zhong, Zongren Li, Xu Chen, Junfeng Wang, Kunlun He

**Affiliations:** ^1^Key Laboratory of Ministry of Industry and Information Technology of Biomedical Engineering and Translational Medicine, Chinese PLA General Hospital, Beijing 100853, China; ^2^Translational Medical Research Center, Chinese PLA General Hospital, Beijing 100853, China; ^3^Medical Artificial Intelligence Research Center, Chinese PLA General Hospital, Beijing 100853, China; ^4^Julius Center for Health Sciences and Primary Care, University Medical Center Utrecht, 3584 CG Utrecht, Netherlands

## Abstract

**Backgrounds:**

Intra-aortic balloon counterpulsation is increasingly used in acute myocardial infarction complicated by cardiogenic shock. The aim of this study was to explore the preference, effect, and prognosis of intra-aortic balloon counterpulsation in acute myocardial infarction complicated by cardiogenic shock patients.

**Methods:**

Data of acute myocardial infarction complicated by cardiogenic shock patients at the Fourth Medical Center of PLA General Hospital were collected retrospectively. A propensity score was calculated with a logistic regression which contained clinically meaningful variables and variables selected by Lasso and then used to match the control group. The cumulative incidence curve and Gray's test were employed to analyse the effect and prognosis of intra-aortic balloon counterpulsation on mortality.

**Results:**

A total of 1962 acute myocardial infarction cases admitted between May 2015 and November 2018 were identified, and 223 cases with acute myocardial infarction complicated by cardiogenic shock were included as the study cohort, which contained 34 cases that received IABP and 189 cases that did not receive IABP. Patients with higher alanine aminotransferase (OR = 1.93, 95% CI 1.29-2.98), higher triglyceride (OR = 3.71, 95% CI 1.87-7.95), and higher blood glucose (OR = 1.08, 95% CI 0.99-1.18) had a higher probability of receiving intra-aortic balloon counterpulsation. In the propensity score matching analysis, 34 cases received intra-aortic balloon counterpulsation and 102 matched controls were included in the comparison. By comparing the cumulative incidence of in-hospital mortality, there was no statistically significant difference between the intra-aortic balloon counterpulsation group and matched control group (*P* = 0.454).

**Conclusion:**

The use of intra-aortic balloon counterpulsation may not improve the prognosis of the acute myocardial infarction complicated by cardiogenic shock patients.

## 1. Introduction

At present, despite marked advances in medical treatment and revascularization techniques, acute myocardial infarction complicated by cardiogenic shock (AMI-cardiogenic shock) has still higher mortality [1–4]. In this urgent clinical treatment situation, one therapeutic selection is intra-aortic balloon pump counterpulsation (IABP), which is the most commonly used intervention for AMI-cardiogenic shock [2, 5–8]. From a pathophysiological point of view, the contributions of IABP treatment include increasing diastolic coronary perfusion and decreasing left ventricular aortic systolic pressure (afterload) and myocardial oxygen consumption [8–15]. Therefore, by reducing the cardiac workload, improving blood flow and cardiac output, IABP is considered a clinical treatment of AMI-cardiogenic shock patients [11]. Some observational studies indicated that the prognosis of patients receiving IABP was better than that of patients not receiving it. The American College of Cardiology and American Heart Association (ACC/AHA) and also the European Society of Cardiology (ESC) strongly recommend the use of IABP in patients with AMI-cardiogenic shock. However, some observational studies and new randomized controlled trials (RCTs) revealed that the use of IABP was not found to improve mortality among AMI patients with cardiogenic shock [5, 12, 14, 16, 17].

Therefore, the use of IABP supports hemodynamics, but evidence on improving the prognosis of AMI-cardiogenic shock patients was still controversial. We established a retrospective cohort study from a single center, to explore the impact of IABP on the mortality of patients with AMI-cardiogenic shock. By matching patients who have received IABP and patients who have not received IABP, we explored the mortality of patients with AMI-cardiogenic shock in the hospital. In addition, we explored the prognostic factors for mortality in AMI-cardiogenic shock patients receiving IABP. Recommendations are given clinically in order to better assist the treatment of AMI-cardiogenic shock and improve the survival rate of patients.

## 2. Methods

### 2.1. Data

A retrospective cohort was established to investigate mortality associated with IABP for acute myocardial infarction complicated by cardiogenic shock. Patients admitted in the Fourth Medical Center of PLA General Hospital between May 2015 and November 2018 with a diagnosis of acute myocardial infarction were identified from the electronic medical records. The exclusion criterion was acute myocardial infarction not complicated by cardiogenic shock.

Demographic, clinical, laboratory, and clinical outcome data were obtained from the hospital's electronic clinical medical records. At the first clinical consultation, demographic, clinical, and laboratory data were collected within 24 hours after admission. The outcome of interest was in-hospital death, with discharge as the competing risk event, and event time was defined as the time from admission to either event whichever came first.

### 2.2. Statistical Analysis

Continuous variables were presented as median and interquartile range (IQR), and categorical variables were presented as number and its corresponding percentage. Variables with a right-skewed distribution were log-transformed before being included in the analysis. Missing values were imputed with single imputation. Univariate logistic regression was used to explore the risk factors of receiving IABP in AMI-cardiogenic shock patients, and a cause-specific Cox model was used to explore the prognostic factors of mortality in AMI-cardiogenic shock patients receiving IABP.

To ensure the assumption of positivity (i.e., each patient has a nonzero probability of being assigned to either treatment group) is met in the propensity score analysis, we used a two-step approach in exploring the predictors of receiving IABP and matching IABP patients with controls.

In the first step, variables that led to (quasi-) complete separation were automatically identified as predictors (with an OR being infinite), and a deterministic matching based on these variables was performed (i.e., patients in the group with zero probability of receiving IABP were excluded from further analyses). In step two, we used Lasso for variable selection to determine other predictors of receiving IABP, after excluding those predictors already identified in step one. The selected variables were used as matching variables (in addition to age and sex) in the propensity score matching analysis (with a ratio of 3 : 1). The number of variables selected by Lasso was determined with the 1-in-10 rule of thumb: number of selected variables = max(1, number of cases/10). The cumulative incidence curve and Gray's test were used to compare the in-hospital mortality of patients receiving IABP and matched controls. Statistical analyses were conducted using R software (version 3.6.1) and packages dplyr, mice, glmnet, Hmisc, AUC, survival, cmprsk, descry, and forestplot.

## 3. Results

### 3.1. Patients

**Figure 1:**
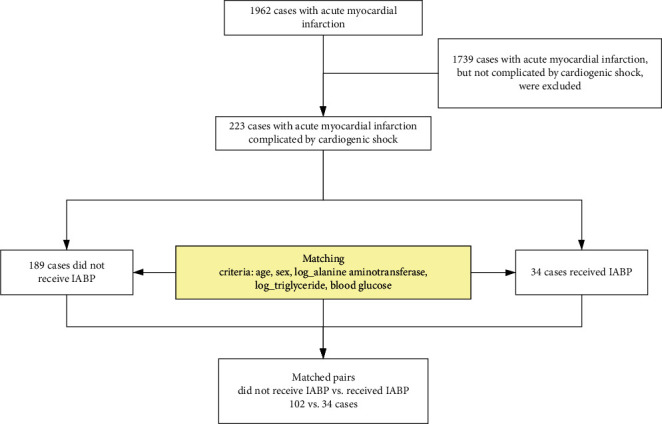
Flowchart of patient inclusion and the matching procedure. From a total of 1962 acute myocardial infarction cases in the Fourth Medical Center of PLA General Hospital, 223 cases met the acute myocardial infarction complicated by cardiogenic shock inclusion and exclusion criteria. Thirty-four cases received IABP, and 102 cases of matched pairs did not receive IABP.

A total of 1962 acute myocardial infarction cases admitted between May 2015 and November 2018 were identified. After excluding 1739 cases not complicated by cardiogenic shock, 223 cases with AMI-cardiogenic shock were included as the study cohort, which contained 34 (15.25%) cases that received IABP and 189 cases that did not receive IABP (Figure 1).

### 3.2. Factors Associated with Receiving IABP

**Figure 2:**
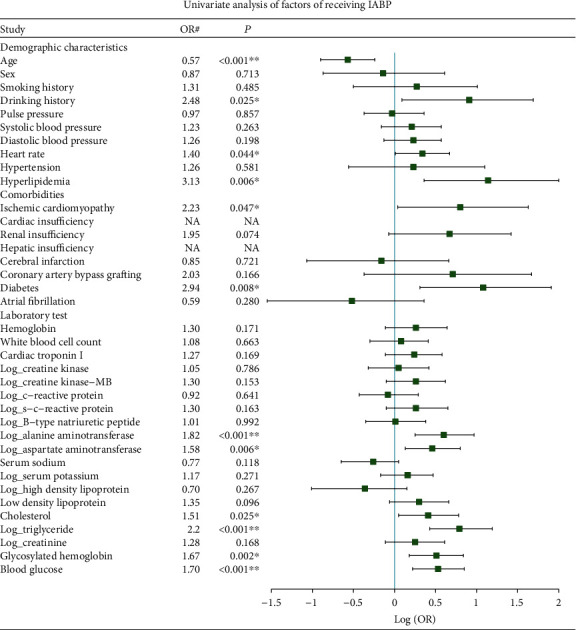
Univariate analysis of factors for receiving IABP in the AMI-cardiogenic shock patients. ^∗^The *P* value is between 0.05 and 0.001. ^∗∗^The *P* value is <0.001. ^#^Standardized OR, OR per SD increase for continuous variables, and OR compared to the reference group for categorical variables. NA: (quasi-) complete separation variables, where OR is not applicable.

Univariate logistic regression revealed that patients with higher drinking history, heart rate, hyperlipidemia, ischemic cardiomyopathy, diabetes, alanine aminotransferase, aspartate aminotransferase, cholesterol, triglyceride, glycosylated hemoglobin, and blood glucose and younger age had a higher probability of receiving IABP (Figure 2). All IABP patients were from cardiac insufficiency and nonhepatic insufficiency groups; thus, these two factors were considered predictive variables for receiving IABP. Next, based on the rule of thumb, three most important variables (34/10) were selected by Lasso: alanine aminotransferase, triglyceride, and blood glucose, and the effect sizes were estimated with a multivariable logistic regression model. Patients with higher alanine aminotransferase (OR = 1.93, 95% CI 1.29-2.98), higher triglyceride (OR = 3.71, 95% CI 1.87-7.95), and higher blood glucose (OR = 1.08, 95% CI 0.99-1.18) had a higher probability of receiving IABP (Table 1).

### 3.3. Effect of IABP

After excluding non-IABP patients in cardiac insufficiency and nonhepatic insufficiency groups, each IABP patient was matched with three controls by the propensity score calculated based on age, sex, alanine aminotransferase, triglyceride, and blood glucose. Finally, 102 patients not receiving IABP were selected as the control group (Figure 1). The distributions of these matching variables were comparable between IABP and matched non-IABP groups (Table 2). The model performance of the propensity score model was good with a C-index of 0.80. Therefore, the model including age, sex, alanine aminotransferase, triglyceride, and blood glucose can provide a reliable prediction on the probability of getting treated with IABP.

**Figure 3:**
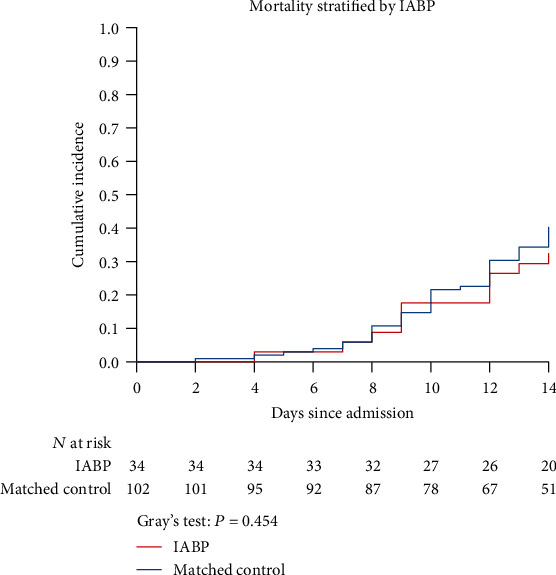
Cumulative incidence curves for in-hospital mortality stratified by IABP. The cumulative incidence was used to assess the primary end point of mortality for the IABP group (red line) and the matched control group (blue line).

When comparing the cumulative incidence of in-hospital mortality, there was no statistically significant difference between the IABP group and the matched control group (*P* = 0.454; Figure 3). The cumulative incidence of mortality at days 7 and 14 was 0.06 (0.00, 0.14) and 0.32 (0.17, 0.24) in patients with IABP, respectively.

### 3.4. Factors Associated with the Mortality in AMI-Cardiogenic Shock Patients Receiving IABP

**Figure 4:**
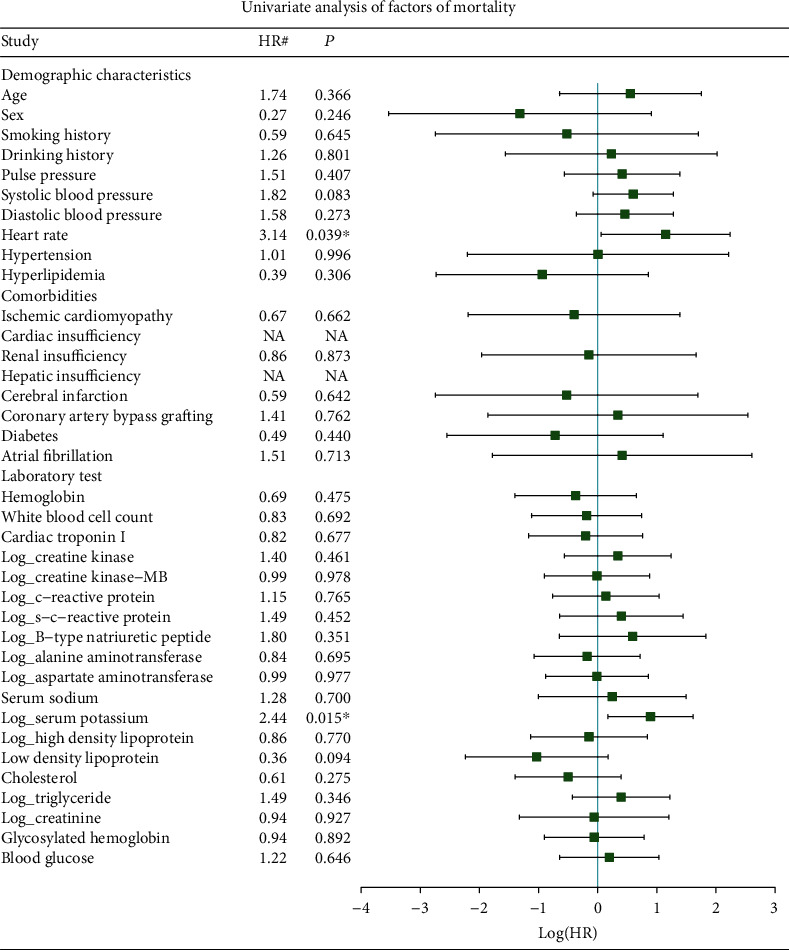
Univariate analysis of factors for mortality in the AMI-cardiogenic shock patients receiving IABP. ^∗^The *P* value is between 0.05 and 0.001. ^∗∗^The *P* value is <0.001; ^#^Standardized HR, HR per SD increase for continuous variables, and HR compared to the reference group for categorical variables. NA: (quasi-) complete separation variables, where HR is not applicable.

Univariate Cox regression revealed that patients receiving IABP with a higher heart rate and serum potassium had a higher probability of death (Figure 4).

## 4. Discussion

This retrospective cohort study revealed that the use of IABP cannot improve the mortality of AMI-cardiogenic shock patients. Despite the fact that there are still great controversies about the use of IABP that can improve the prognosis of AMI-cardiogenic shock patients and some observational studies revealed a trend toward lower mortality for patients if they received IABP, these findings may result from the clear inequality of baseline risk factors [18, 19]. In our study, we used Lasso regression to identify the most important factors of receiving IABP and used them as the matching parameters (in addition to age and sex) to balance the baseline risk factors. From the cumulative incidence curve of AMI-cardiogenic shock mortality stratified by IABP use, there was no statistically significant difference in 14-day mortality between the IABP group and matched control group. Our study result is consistent with the conclusions of some observational and RCT studies [12, 13, 20–23]. Therefore, IABP may not improve the prognosis of AMI-cardiogenic shock patients.

From a pathophysiological point of view, the main feature of death in AMI-cardiogenic shock is unstable hemodynamics with reduced systolic and mean arterial pressures that lead to reduced oxygen supply to vital organs [9], while the IABP can give hemodynamic support to these hemodynamically unstable patients by increasing blood flow to the heart and decreasing the cardiac workload [2]. However, the reason why the mortality of AMI-cardiogenic shock patients was not sufficiently reduced may be because the effects on cardiac output are modest [24]. Therefore, we speculate that the therapeutic effects seem to be limited in improving hemodynamics, which apparently could not be converted into an improved prognosis of AMI-cardiogenic shock patients.

Our results revealed that higher heart rate and serum potassium may lead to higher mortality in AMI-cardiogenic shock patients receiving IABP. A previous study revealed that serum potassium and heart rate can increase the risk of mortality of patients with AMI [25]. Therefore, heart rate and serum potassium need to be closely monitored for AMI-cardiogenic shock patients receiving IABP.

Our study has several limitations. First, the sample size of our study is not large enough. Therefore, the research may not have good statistical power, which may be one of the reasons why statistically significant result was not observed. Therefore, a larger cohort is needed to further study the effect of IABP on the mortality of patients with acute myocardial infarction complicated by cardiogenic shock. Second, this is a retrospective single-center study, and some laboratory tests were not taken within the first day after admission. We used an imputation method to deal with these missing values; however, a single imputation may lead to uncertainty in results. A prospective cohort will provide stronger evidence.

In conclusion, the use of intra-aortic balloon counterpulsation may not improve the prognosis of the acute myocardial infarction complicated by cardiogenic shock patients.

## Figures and Tables

**Table 1:** Multivariate logistic regression for receiving IABP in the AMI-cardiogenic shock patients.

Variable	OR (95% CI)	*P*
Log (alanine aminotransferase)	1.93 (1.29, 2.98)	<0.001^∗∗^
Log (triglyceride)	3.71 (1.87, 7.95)	0.001^∗^
Blood glucose	1.08 (0.99, 1.18)	0.085

^∗^The *P* value is between 0.05 and 0.001. ^∗∗^The *P* value is <0.001.

**Table 2:** Clinical characteristics of the matched patients (the IABP group and the matched control group) in the Fourth Medical Center of PLA General Hospital.

Variable^#^	IABP group (*N* = 34)	Matched control group (*N* = 102)
Age	66.00 (62.00, 82.25)	77.00 (67.75, 81.00)
Sex (male)	19 (55.90%)	57 (55.90%)
Log (alanine aminotransferase)	3.71 (2.69, 4.24)	3.18 (2.76, 3.91)
Log (triglyceride)	0.47 (0.02, 0.91)	0.16 (-0.07, 0.37)
Blood glucose	8.78 (5.96, 15.55)	7.41 (5.32, 9.96)

^#^Continuous variable (median, IQR); categorical variable (*N*, percent).

## Data Availability

The research data used to support the findings of this study are available from the corresponding authors upon request.

## Supplementary Materials

Supplementary Materials Supplemental Table 1: clinical characteristics of the AMI-cardiogenic shock patients.
