# Supplementary material for: The Preference, Effect, and Prognosis of Intra-Aortic Balloon Counterpulsation in Acute Myocardial Infarction Complicated by Cardiogenic Shock Patients: A Retrospective Cohort Study
Source: Biomed Res Int. 2021 Jan 20;2021:6656926. doi: 10.1155/2021/6656926 (PMC7840249; doi:10.1155/2021/6656926)
Supplement: Supplementary Materials — Supplemental Table 1: clinical characteristics of the AMI-cardiogenic shock patients. [file 6656926.f1.docx]

Supplemental Table 1: Clinical Characteristics of the AMI-cardiogenic shock patients.

|  | Patients received IABP (N=34) | Patients did not receive IABP (N=189) |
| --- | --- | --- |
| **Demographic characteristics** |  |  |
| Age | 66.00(62.00,81.50) | 78.00(72.00,84.00) |
| Sex | 19(55.88) | 19(10.22) |
| Smoking history | 14(41.18) | 14(7.53) |
| Drinking history | 12(35.29) | 12(6.45) |
| Pulse pressure | 53.00(46.00,64.00) | 52..50(42.00,64.00) |
| Systolic blood pressure | 120.00(112.00,130.00) | 115.00(102.00,131.25) |
| Diastolic blood pressure | 68.00(61.00,74.00) | 59.00(54.00,72.00) |
| Heart rate | 86.00(80.00,103.00) | 76.00(68.00,89.00) |
| Hypertension | 24(70.59) | 24(12.90) |
| Hyperlipemia | 25(73.53) | 25(13.44) |
| **Combordities** |  |  |
| Ischemic cardiomyopathy | 11(32.35) | 11(5.91) |
| Cardiac insufficiency | 33(97.06) | 33(17.74) |
| Renal insufficiency | 18(52.94) | 18(9.68) |
| Hepatic insufficiency | 0(0.00) | 0(0.00) |
| Cerebral infarction | 8(23.53) | 8(4.30) |
| Coronary artery bypass grafting | 5(14.71) | 5(2.69) |
| Diabetes | 24(70.59) | 24(12.90) |
| Atrial fibrillation | 6(17.65) | 6(3.23) |
| **Laboratory test** |  |  |
| Hemoglobin | 121.00(101.00,138.00) | 116.00(95.00,130.00) |
| White blood cell count | 9.10(7.60,13.40) | 8.40(6.17,11.80) |
| Cardiac troponin I | 2.00(0.41,43.00) | 0.99(0.02,9.02) |
| Creatine kinase | 176.00(49.00,1326.00) | 161.00(69.50,703.00) |
| Creatine kinase-MB | 30.00(11.10,121.00) | 17.00(10.00,53.00) |
| C-reactive protein | 62.00(28.06,125.85) | 63.70(20.00,95.40) |
| S-c-reactive protein | 29.75(7.60,106.42) | 23.65(5.53,63.42) |
| B-type natriuretic peptide | 6510.00(2960.00,15154.25) | 7005.00(2445.00,18842.00) |
| Alanine aminotransferase | 37.00(15.00,65.00) | 19.00(11.00,32.50) |
| Aspartate aminotransferase | 48.00(22.00,165.00) | 27.00(19.00,74.00) |
| Serum sodium | 140.00(139.00,142.00) | 141.00(137.00,142.00) |
| Serum potassium | 4.11(3.71,4.36) | 3.95(3.67,4.45) |
| High density lipoprotein | 0.93(0.74,1.09) | 1.03(0.85,1.28) |
| Low density lipoprotein | 2.26(1.74,2.81) | 1.93(1.50,2.75) |
| Cholesterol | 3.95(3.48,4.57) | 3.42(2.82,4.35) |
| Triglyceride | 1.64(1.14,2.33) | 0.97(0.69,1.34) |
| Creatinine | 98.00(71.00,187.00) | 86.00(64.00,120.10) |
| Glycosylated hemoglobin | 7.20(5.65,8.40) | 6.40(5.80,7.30) |
| Blood glucose | 9.10(6.10,15.54) | 6.43(5.13,8.73) |

^#^continuous variable (median, IQR); categorical variable (N, percent).
